# Expanding Newborn Screening for Pompe Disease in the United States: The NewSTEPs New Disorders Implementation Project, a Resource for New Disorder Implementation

**DOI:** 10.3390/ijns6020048

**Published:** 2020-06-11

**Authors:** Kshea Hale, Yvonne Kellar-Guenther, Sarah McKasson, Sikha Singh, Jelili Ojodu

**Affiliations:** 1 Association of Public Health Laboratories, Silver Spring, MD 20910, USA; sikha.singh@aphl.org (S.S.); jelili.ojodu@aphl.org (J.O.); 2 Center for Public Health Innovation, Littleton, CO 80120, USA; ykellar-guenther@ciinternational.com; 3 Colorado School of Public Health, Department of Epidemiology, University of Colorado, Anschutz Medical Campus, Aurora, CO 80045, USA; sarah.mckasson@cuanschutz.edu

**Keywords:** newborn screening, Pompe disease, new disorders implementation

## Abstract

Public health programs in the United States screen more than four million babies each year for at least 30 genetic disorders. The Health and Human Services (HHS) Advisory Committee on Heritable Disorders in Newborns and Children (ACHDNC) recommends the disorders for state newborn screening (NBS) programs to screen. ACHDNC updated the Recommended Uniform Screening Panel (RUSP) to include Pompe disease in March 2015. To support the expansion of screening for Pompe disease, the Association of Public Health Laboratories (APHL) proposed the Newborn Screening Technical assistance and Evaluation Program (NewSTEPs) New Disorders Implementation Project, funded by the HHS’ Health Resources and Services Administration (HRSA) Maternal and Child Health Bureau (MCHB). Through this project, APHL provided financial support to 15 state NBS programs to enable full implementation of screening for Pompe disease. As of April 27, 2020, nine of the 15 programs had fully implemented Pompe disease newborn screening and six programs are currently pursuing implementation. This article will discuss how states advanced to statewide implementation of screening for Pompe disease, the challenges associated with implementing screening for this condition, the lessons learned during the project, and recommendations for implementing screening for Pompe disease.

## 1. Introduction

Public health programs in the United States screen more than four million babies each year for at least 30 genetic disorders. Through the newborn screening (NBS) program, states identify newborns at risk for certain metabolic, endocrine, hemoglobin, neuromuscular, and other inherited conditions. To guide this process, the Health and Human Services (HHS) Advisory Committee on Heritable Disorders in Newborns and Children (ACHDNC) recommends the disorders for state NBS programs to screen [1]. Each disorder is selected through a vigorous evidence review process. Once approved, these disorders are included on the Recommended Uniform Screening Panel (RUSP).

There are 35 core disorders on the RUSP, as of April 2020 [2]. ACHDNC updated the RUSP to include Pompe disease in March 2015.

Pompe disease is a multi-systemic lysosomal storage disorder (LSD) caused by a deficiency in the acid alpha glucosidase (GAA) enzyme. This enzyme helps lysosomes breakdown glycogen, a complex sugar, in the body. Without this enzyme, glycogen accumulates in the cells, causing them to lose their ability to function properly. If left untreated, babies born with the most severe form of Pompe disease die within their first year of life [3]. In the United States, Pompe disease affects approximately one in 40,000 births. Implementing NBS for Pompe disease allows babies with the disorder to be identified and linked to care, often before symptoms arise. As of April 2020, 23 states in the US screen for Pompe disease, nine of which received direct funding and technical assistance from the Association of Public Health Laboratories (APHL) to implement screening for Pompe disease.

To implement universal screening for Pompe disease, there are many factors for state NBS programs to consider. This includes determining the cost to screen for the disorder, gaining legislative and funding approval to screen, securing fee increases if necessary, selecting a technology and methodology to screen, expanding laboratory space and capacity, acquiring the appropriate equipment, hiring laboratory and follow-up staff, and developing and implementing education initiatives for the public and for clinical providers. While these factors are important for the addition of all new disorders to state screening panels, Pompe disease introduced unique considerations for the newborn screening system. States screening for this disorder must consider establishing algorithms for the detection of infantile Pompe disease in distinction from late onset Pompe disease, defining the duration of short term follow up and late onset Pompe disease and defining the role of NBS in long term follow up (LTFU) for individuals identified with the disorder. 

## 2. Methods 

APHL was awarded a cooperative agreement from the HHS’ Health Resources and Services Administration (HRSA) Maternal and Child Health Bureau (MCHB) to expand the ability of state NBS programs to screen for Pompe disease. Through the New Disorders Implementation Project, the APHL Newborn Screening Technical assistance and Evaluation Program (NewSTEPs) provided financial support over the course of three years to 15 state NBS programs to enable and accelerate full implementation of newborn screening for Pompe disease [4].

**Figure 1:**
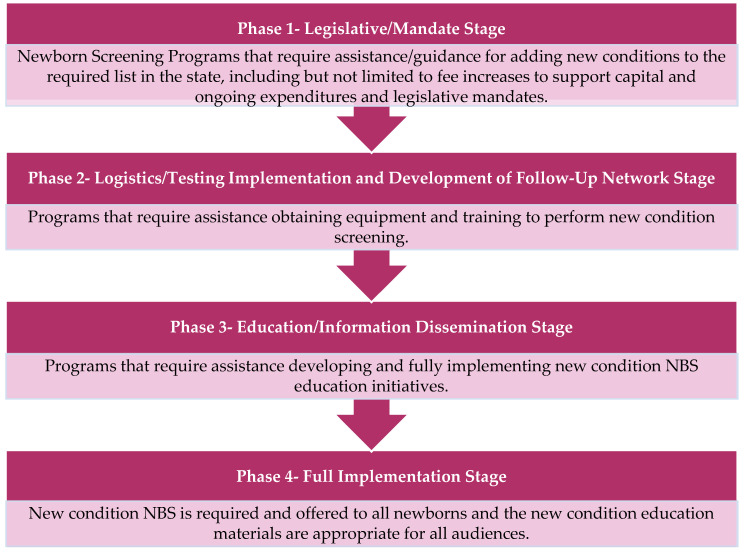
Phases of Implementation for New Disorders Newborn Screening.

APHL also selected and funded three additional states to serve as Peer Network Resources Centers (PNRCs) during the course of the New Disorders Implementation Project. The PNRCs served as content area experts providing technical assistance and training regarding laboratory techniques, follow-up procedures, and educational processes related to Pompe disease, as well as for other new disorders screening as needed. Each state NBS program and PNRC applied to participate in the project through a competitive request for proposals (RFP) and a subsequent evaluation process. In the RFP, NewSTEPs proposed a four-phase model to stratify the NBS implementation process, as seen in Figure 1 [5]. In their RFP response, each state program indicated where they were in the implementation of screening for Pompe disease, which other new disorders they were seeking support for, what they hoped to accomplish with this funding opportunity, and how they would use the funding. 

The 15 participating state NBS programs provided a combination of monthly and annual progress reports where they described activities and progress toward full implementation and also participated in monthly calls with NewSTEPs staff to track and describe their activities and indicate their successes, challenges, needs, lessons learned, and recommendations. 

## 3. Results

Of the 15 funded state newborn screening programs, nine were also funded to implement screening for Mucopolysaccharidosis type I (MPS I) and X-linked adrenoleukodystrophy (X-ALD) simultaneously with Pompe disease and six were funded to implement MPS I simultaneously with Pompe disease. 

Additionally, the 15 funded programs set varied project goals and objectives to move them toward full implementation of screening for Pompe disease during the funding period. A summary of these goals are found in Table 1.

### 3.1. Progress toward Pompe Disease NBS Implementation

Upon initiation of the funded project, two of the 15 state NBS programs were working on Phase 1 (Figure 1) to obtain approval and secure fee increases from their NBS Advisory Committee, Board of Health Commissioner, and/or State Budget Authority to screen for Pompe disease.

Upon initiation of the funding project, nine state NBS programs reported that they were working on Phase 2 (Figure 1) toward ensuring infrastructure and staffing readiness for Pompe disease newborn screening. Activities during this time included identifying screening methodologies, acquiring equipment, developing algorithms and protocols for screening, determining cut-off values for analysis, establishing contracts with medical specialists and treatment centers, developing long-term follow-up programs, pursuing assay validation, and integrating Pompe disease reporting features into their Laboratory Information Management Systems (LIMS).

Upon initiation of funding, three state NBS programs reported that they would be working toward Phase 2 (Figure 1) in securing infrastructure and staffing readiness simultaneously with Phase 3 (Figure 1) in developing educational materials for providers, families, and the general public as well as implementing educational and outreach activities.

Upon initiation of funding, only one state NBS program reported that they would be engaging in Phase 1, Phase 2, and Phase 3 (Figure 1) simultaneously.

In their applications, 10 state NBS programs anticipated that they would have implemented Pompe disease screening at the end of the project period, one program anticipated that they would have entered Phase 3 (Figure 1), and two programs anticipated that they would have entered Phase 2 (Figure 1) at the conclusion of the funding period.

### 3.2. How States Advanced to Statewide Implementation of Screening for Pompe Disease

Participating state NBS programs utilized funding from the NewSTEPs New Disorder Implementation Project to purchase equipment and supplies, develop and translate educational materials, develop a statewide outreach program, host a community engagement event, hire laboratory and follow-up personnel, purchase software for sequence analysis, travel to professional meetings or to PNRCs for training, configure LSDs workflow in Laboratory Information Management Systems (LIMS), and develop a long-term follow-up program (Table 2).

Each program engaged at least one PNRC for technical assistance during the project. The PNRCs provided states with LSD standard operating procedures and algorithms, sample panels for validation, technical assistance around LSD test validation, LSD cost data, comparison analysis, second-tier testing guidance for Pompe disease, DNA sequence analyses for Pompe disease, and Pompe disease related educational materials for primary care providers and parents. The PNRCs also retested abnormal LSDs for state programs. One PNRC hosted an annual Tandem Mass Spectrometry, DNA Sequencing, and a Follow-up hands-on workshop. Another program prepared Pompe disease follow-up flow diagrams. State NBS programs also visited PNRCs for direct one-on-one-training to learn laboratory and follow up best practices and to observe processes.

As of 27 April, 2020, nine of the 15 programs that participated in the project had fully implemented Pompe disease newborn screening, offering the screening universally to all newborns in the following states: California, Michigan, Minnesota, Nebraska, New Jersey, Ohio, Tennessee, Virginia, and Washington. The other six programs are currently pursuing screening implementation for Pompe disease.

Programs participating in the NewSTEPs New Disorders Implementation project to implement Pompe Disease Newborn Screening also achieved the following milestones during the course of the funding period: created and deployed an online education module for providers and parents, educated 500 health care professional across the state regarding Pompe disease, implemented second-tier testing for Pompe disease, developed a follow-up quality improvement plan, and hosted a successful deliberative community engagement event.

It is important to note that while the activities for the 15 state newborn screening programs highlighted here are specific to their pursuit of Pompe disease newborn screening, additional disorders (MPS I and x-ALD) were also pursued by a number of these programs. There are several aspects of the newborn screening multiplexing of the screening assay, development of educational materials, and training of implementation activities described above that are generalizable to these other disorders, including laboratory and follow-up staff, establishment of reporting algorithms, among others.

## 4. Challenges

The participating state NBS programs faced several challenges implementing screening for Pompe disease. As noted above, while many barriers and challenges in implementing newborn screening are generalizable to multiple disorders, the challenges presented below are articulated by the 15 newborn screening programs pursuing Pompe disease newborn screening during the course of the NewSTEPs New Disorders Implementation Project. These challenges are as follows (Table 3):

## 5. Lessons Learned

When implementing screening for Pompe disease the participating state newborn screening programs recommended allowing for sufficient time to accomplish procurement of instrumentation required for first tier detection of GAA enzyme activity. Multiple methodologies exist to perform a Pompe disease newborn screening assay, including the use of digital microfluidics or tandem mass spectrometry, and programs recommended building in sufficient time to identify the methodology to use [6]. In addition, delays should be anticipated when performing building and space modifications to accommodate Pompe disease newborn screening into the existing workflow. In order to address these delays associated with introducing Pompe disease newborn screening, while maintaining existing quality practices, adequate staffing should be available. 

Before implementing full population screening for Pompe disease, the participating states noted that NBS programs should determine second-tier needs, address special populations since these infants can potentially influence the determination of cut-off values for assay analysis and may require repeat screening, and ensure LIMS integration is complete. The introduction of Pompe disease newborn screening can increase the workload for all staff involved in the testing and reporting process, therefore affecting communication with providers. 

## 6. Recommendations 

At the end of the NewSTEPs New Disorder Implementation Project, the participating state NBS programs provided recommendations for implementing newborn screening for Pompe disease. The recommendations included participating in APHL NewSTEPs committees and in-person meetings to collaborate and connect with other programs and NBS stakeholders, creating standardized educational materials and long-term follow-up strategies that can be modified for state-specific use, and ensuring that all state stakeholders including pediatric subspecialties and parent representatives are involved in discussing all plans for implementation. Other recommendations include adding second-tier screening to reduce false positives and monitoring screening outcomes and refining cut-off reporting algorithms. Prior to implementing second-tier screening or refining cut-offs, states’ programs screening for Pompe disease using tandem mass spectrometry or digital microfluidics reported higher than expected false positive and referral rates, increasing the patient load at NBS follow-up coordinating and genetic centers. Implementing second-tier testing and refining cut-offs reduced false positive and referral rates. Participating state NBS programs also recommended developing long-term follow-up guidelines for cases of Late Onset Pompe Disease (LOPD). 

## 7. Conclusions

The ACHDNC updated the RUSP to include Pompe disease in March 2015. To support the expansion of newborn screening across the United States for Pompe disease, APHL proposed the NewSTEPs New Disorders Implementation Project, funded by HRSA. Through this project, 15 state NBS programs received direct funding through a competitive RFP process to implement screening for Pompe disease and all states had access to technical assistance and training via PNRCs. To provide further support to state NBS programs with the implementation of Pompe disease, APHL hosted national meetings to provide a platform for state programs to share their experiences implementing screening, hosted disease-specific webinars, and convened a NewSTEPs New Disorders Workgroup to serve as educational experts. As a result of the project, all states had access to information and resources regarding Pompe disease screening implementation.

To ensure that all states have the capacity to screen for Pompe disease and newborns living with this disorder are identified and linked to care, APHL will continue to provide support through technical assistance, training, webinars, quality improvement initiatives, and national meetings.

## Figures and Tables

**Table 1:** Goals being pursued by the 15 Association of Public Health Laboratories (APHL) funded states for Pompe disease newborn screening implementation.

Project Goals for Pompe Disease Implementation	Number of States (% of Total, *n* = 15)
Full implementation of population screening	5 (33%)
Implementation of education or follow-up activities	4 (27%)
Validation activities and pilot screening	6 (40%)

**Table 2:** Utilization of funding by states to implement screening for Pompe disease.

Project Expenses	Number of States (*n* = 15)
Equipment and supplies	2
Staffing	1
Educational activities	0
Travel	0
Other expenses	0
Equipment, supplies, and travel	2
Equipment, supplies, and educational activities	1
Staffing, travel, educational activities, and other expenses	2
Equipment, supplies, staffing, educational activities, and other expenses	2
Equipment, supplies, staffing, travel, educational activities, and other expenses	3
Equipment, supplies, travel, educational activities, and other expenses	1
Equipment, supplies, staffing, and other expenses	1

**Table 3:** Challenges reported by participating state newborn screening programs when implementing Pompe Disease Newborn Screening during the course of the NewSTEPs New Disorder Implementation Project.

Challenge	Reason Provided
Hiring	Lengthy in-state human resources approval processes
	Lack of qualified applicants to perform enzyme activity detection and/or genetic testing
	Lack of qualified applicants to analyze results and perform risk assessments for the various stages of Pompe disease onset
Retention of Staff	Availability of more lucrative positions elsewhere
	Staff retirements coupled with lack of backfilling these vacancies
Procurement Delays	Complicated and time-consuming state procurement processes
	Delays by the state procurement office in establishing contracts with vendors to purchase reagents and lease instrumentation to perform the assay for Pompe disease
Instrumentation Challenges	New instrumentation did not perform as expected
Infrastructure Challenges	Delays in the construction of adequate laboratory space to house instrumentation required for the addition of Pompe disease newborn screening
Information Technology	Delays by vendors to complete upgrades to Laboratory Information Management Systems (LIMS) to accommodate results reporting and messaging for newly added disorder (Pompe disease) to the workflow
	Time and expense involved in gathering system requirements and working with Information Technology (IT) developers and informatics support to modify LIMS and vendor specific software
Clinical Follow-up	The pediatric community and other healthcare providers who would be responsible for caring for babies with an abnormal result have limited knowledge regarding Pompe disease
